# Reflex anuria following acute cardiac event

**DOI:** 10.1186/1471-2369-14-106

**Published:** 2013-05-20

**Authors:** Vijayabala Jeevagan, Mitrakrishnan Navinan, Arunie Munasinghe, Abdul Nazar, Anura Wijewardena, Godwin Constantine

**Affiliations:** 1 University Medical Unit, National Hospital of Sri Lanka, Colombo-5, Sri Lanka; 2 Asiri Hospital, Colombo, Sri Lanka

## Abstract

**Background:**

Reflex anuria is an uncommon cause for acute renal failure, which occurs almost always after manipulation or irritation to kidneys, ureter, bladder or other pelvic organs.

**Case presentation:**

Here we describe a case of acute renal failure due to reflex anuria following acute cardiac event. This patient had background history of urolithiasis. In the absence of other pre renal, renal or post- renal causes for acute kidney injury, we believe reflex anuria is the causative entity for acute renal failure in our patient.

**Conclusion:**

Acute renal failure due to reflex anuria is related to a reflex mechanism involving arteriolar vasoconstriction and urethral spasm. Patients with reflex anuria can be successfully managed with medical or surgical interventions. Our case suggests that reflex anuria should be considered as one of the differential diagnosis of acute renal failure following acute cardiac event, especially in patients with background urological problem.

## Background

In unilateral ureteric obstruction renal function remains normal or only mildly impaired due to the compensation of the normally functioning contralateral kidney. In the medical literature, there have been few reports of acute renal failure (ARF) with unilateral ureteric obstruction and normally functioning contralateral kidney [1-3]. This rare entity is described as reflex anuria(RA). Even more rarely, RA with ARF had also been described without organic obstruction and is thought to be due to manipulation and neural irritation of pelvic organs [4,5].

RA is defined as “cessation of urine output from both kidneys in response to irritation or trauma to one kidney or its ureter or severely painful stimuli to other pelvic organs [1,3,4]”. The diagnosis of RA is based on three criteria [6]:

1. A normal contralateral kidney, which retains normal function soon after the disease causing non-function of the other kidney, has been treated.

2. Subsequent investigation of the contralateral kidney shows that a pathological process is unlikely to have caused its loss of function.

3. Surgical intervention to the contralateral shut down kidney does not result in return of function in either kidney.

Here we report a case of acute renal failure (ARF) due to reflex anuria (RA) precipitated by acute cardiac event. Most of the reference text books do not consider RA as a differential diagnosis for ARF. To our knowledge, ARF due to RA following acute cardiac event has not been reported.

## Case presentation

A 50 year old male presented with intermittent colicky abdominal pain for 3 months. Abdominal ultrasound and the KUB (Kidney, ureter and bladder) x-ray revealed 1.2 cm calculus in the mid right ureter with mild hydronephrosis. His left kidney was normal in size and both kidneys showed normal corticomedullary pattern. His renal function was normal.

While waiting for lithotripsy he developed chest pain which turned out to be extensive anterior ST elevation myocardial infarction. Coronary angiography showed a complete occlusion of the left anterior descending artery (LAD). He underwent primary angioplasty to the proximal LAD, 150 mL of iodixanol contrast agent was used for this intervention. His haemodynamic parameters remained stable throughout. He was normotensive. His jugular venous pressure was not elevated and the lung bases were clear. From the onset of chest pain he did not pass any urine. Anuria persisted despite adequate fluid resuscitation. He was treated with 80 mg of intravenous furosemide without any response.

His full blood count was normal and there was no eosinophilia. Urine full report was normal without any urine eosinophils. Serum complement (C3 and C4) level was normal. However his creatinine continued to rise steadily, and reached 6.2 mg/dl at 48 hours after the stenting. Urine electrolyte value and fractional excretion of sodium were not consistent with a pre-renal pathology. Repeatedly abdominal ultrasound scan with Doppler showed right ureteric calculus and failed to reveal any abnormalities in renal arterial or venous flow. 2D echo cardiogram showed anterior wall hypokinesia with preserved ejection fraction. Since his renal function deteriorated, he was supported with haemodyalysis. Total anuria persisted for three days. On day four he passed 30 ml of urine. Six days after myocardial infarction he underwent ureteric stent implantation to the right ureter. Despite the stenting, he was anuric (i.e. <50 ml/day) until the day nine. His urine output gradually improved thereafter. Two months after the acute event he underwent successful lithotripsy. After 6 months of the acute event his DTPA scan showed normal renal function.

## Discussion

Here we have described a case of anuria and acute renal failure precipitated by acute cardiac event. Extensive clinical evaluation failed to reveal any prerenal, renal or postrenal etiologies as the cause for ARF. Our patient had developed anuria abruptly at the onset of acute cardiac event. In the absence of other causes for ARF and development of abrupt anuria in the background of unilateral ureteric stone, we believe RA is the causative entity for ARF in our patient.

One might argue that the contrast nephropathy was responsible for this patient’s renal failure. However our patient didn’t have any major risk factors for contrast nephropathy such as renal impairment or diabetes mellitus. Furthermore, he was given only a small amount nonionic low osmolar contrast. Therefore the risk of contrast induced renal failure in our patient is negligible. Our patient was anuric even before the contrast was given. Contrast nephropathy peaks after 12 to 24 hours and is usually nonoliguric [7]. Anuria can only occur in established renal failure. Total anuria from the outset is not compatible with contrast nephropathy. Therefore the rapid development of anuria without any risk factors makes contrast nephropathy an unlikely etiology in this patient.

The challenge is to understand the pathophysiological mechanism responsible for this complex syndrome. There are various mechanisms that have been proposed to explain RA. Hull et al. postulated two reflex mechanisms to explain RA; neurovascular reflex resulting in intense intrarenal vasospasm and bilateral ureteric spasm secondary to unilateral ureteric or renal parenchymal damage where both are associated with pain [1]. There are several examples of experimental support for this neurovascular hypothesis. Di Salvo and Fell were able to demonstrate cessation of renal blood supply by using pulsatile renal nerve stimulation [8]. It is not clear why patients with unilateral urolithiasis are predisposed to this neurovascular reflex. We postulate that in these patients prolonged irritation of a ureter reduces the threshold for the autonomic nervous system to visceral stimuli.

In our patient background urolithiasis would have increased the sensitivity of autonomic nervous system to visceral stimuli. Sympathetic activation of heart due to acute myocardial infarction or coronary intervention may have activated the cardio renal reflex. Because of the complex interaction between kidneys and heart, activation of cardiac sympathetic fibers would have stimulated the activity of renal sympathetic nerves which resulted in reflex intrarenal vasoconstriction and bilateral ureteral spasm.

Patients with reflex anuria can be successfully managed with medical or surgical interventions. Supportive care includes renal replacement therapy, blood pressure control and optimal management of fluid and electrolyte imbalance. There are case reports of successful management of RA by bilateral ureteric stent insertion [9]. The stents should be removed in a graded manner when renal function is normalized to prevent recurrence.

## Conclusion

In cardiology practice, it is common to encounter patients with ARF. However uncommon it may be, RA should be considered as one of the differential diagnosis of ARF following acute cardiac event. This rare condition must be differentiated from other causes of ARF by proper clinical evaluation. Because of the complex nature of this syndrome, it is important that cardiologists, nephrologists and internists should work together towards the common goal of protecting the patient by using best management available based on evidence.

## Consent

Written informed consent was obtained from the patient for publication of this case report. A copy of the written consent is available for review by the Editor-in-Chief of this journal.

## Competing interests

The authors declare that they have no competing interests.

## Authors’ contributions

VJ and GC did the literature survey and wrote the manuscript. All authors involved in the management of the patient. All authors read and approved the final manuscript.

## Pre-publication history

The pre-publication history for this paper can be accessed here:

http://www.biomedcentral.com/1471-2369/14/106/prepub
